# Cropsi study: Efficacy and safety of cryotherapy and cryocompression in the prevention of chemotherapy-induced peripheral neuropathy in patients with breast and gynecological cancer–A prospective, randomized trial

**DOI:** 10.1016/j.breast.2024.103763

**Published:** 2024-06-26

**Authors:** Christine Brunner, Miriam Emmelheinz, Daniel Egle, Magdalena Ritter, Katharina Leitner, Verena Wieser, Carmen Albertini, Samira Abdel Azim, Irene Mutz-Dehbalaie, Johanna Kögl, Christian Marth

**Affiliations:** Department of Obstetrics and Gynecology, Medical University of Innsbruck, Innsbruck, Austria

**Keywords:** Breast cancer, Chemotherapy, Chemotherapy-induced peripheral neuropathy, Cryotherapy, Cryocompression

## Abstract

**Objective:**

This study aimed to demonstrate the superiority of cryocompression over cryotherapy alone in the prevention of chemotherapy-induced peripheral neuropathy (CIPN) grade 2 or above.

**Methods:**

This prospective randomized study was conducted between May 2020 and January 2023 in Innsbruck. Eligible patients had a diagnosis of gynecological cancer and received a minimum of 3 cycles of taxane-based CT (neoadjuvant, adjuvant or palliative therapy). Patients were randomized 1:1 to receive either cryotherapy or cryocompression on their upper extremities during chemotherapy (CT). We performed temperature measurements, two QoL questionnaires and neurological tests during CT and at follow-up 3 and 6–9 months after the completion of CT. CIPN was assessed using the CTCAE score.

**Results:**

Of 200 patients recruited, both groups showed a lower prevalence of CIPN in this study compared to recent literature. In the group receiving cryotherapy, the prevalence of grade 1 CIPN was 30.1 %, and that of grade 2 CIPN or above was 13.7 %; in the group treated with cryocompression, the prevalence of grade 1 CIPN was 32.8 %, and that of grade 2 or above CIPN was 17.2 %. We found a significant reduction in temperature in the cryotherapy and cryocompression groups. Regarding the two QOL questionnaires as well as the neurological tests no significant differences were found between the two groups.

**Conclusion:**

Our study suggests that cryotherapy as well as cryocompression is a safe and effective way to cool patients’ extremities to lower the prevalence of CIPN. Cryocompression was not more effective than cryotherapy alone in the prevention of CIPN.

## Highlights

• Patients receiving cryotherapy or cryocompression developed remarkably rarely CIPN compared to recent literature.
• No severe side effects were observed using cryotherapy as well as cryocompression.
• Cryotherapy and cryocompression achieved a significant reduction in temperature.
• During follow-up a “coasting” phenomenon was observed.

## 1 Background

Cancer ranks as one of the leading causes of death. Therefore, a substantial number of patients are affected by consequences of its treatment [1]. Constant advancements in cancer therapy have resulted in a drastic improvement in overall survival for patients with gynecological cancers. Thus, the impact on patients’ quality of life (QoL) as well as the long-term effects of therapy are gaining importance in treatment planning.

Taxane-based CT is state-of-the-art treatment for many cancers, especially breast and other gynecological cancers [2,3]. One of the side effects of taxane-based CT is chemotherapy-induced peripheral neuropathy (CIPN), which can heavily impact patients’ QoL [[4], [5], [6]]. CIPN is a common treatment-related adverse effect that necessitates dose reductions or early discontinuation of CT [4,5,7]. Individuals suffer from sensory and motor deficits, which are dose-dependent and distributed symmetrically in a “glove and stocking” manner [8,9]. Symptoms include severe pain, numbness, nail changes and a tingling sensation in the fingertips and toe tips [10]. CIPN has been linked to depression, anxiety and poor sleep quality [11]. According to the MASCC (Multinational Association of Supportive Care in Cancer) there is currently no effective approach for CIPN prevention using drugs [12].

Further small studies reported successful prevention of CIPN with cryotherapy as well as cryocompression [[13], [14], [15], [16], [17]]. Bandla et al. found that adding compression to cryotherapy might increase the preventative effect, as cryotherapy leads to a vasoconstriction-driven neuroprotection and this may be enhanced by adding compression [17].

The aim of our randomized study was to evaluate whether compression added to cryotherapy could improve the efficacy of cryotherapy in the prevention of CIPN grade 2 or above. Compression alone was not used as the current literature reports mixed results regarding its' efficacy in preventing CIPN. A study by Kotani et al. did not show a significant benefit of using surgical gloves to prevent CIPN [18]. To precisely assess CIPN in this prospective randomized study, we used QoL questionnaires, and neurological tests. Additionally, we documented temperature changes, to evaluate if one method was more effective in cooling patients’ extremities. We also assessed nail changes as they often affect patients in the long term.

## 2 Methods

### 2.1 Study design

This prospective randomized study was conducted between May 2020 and January 2023 at the Department of Obstetrics and Gynecology at the Medical University of Innsbruck. We performed a wide range of tests and assessments five times during treatment and follow-up, which lasted up to 9 months. Tests included temperature measurements, two QoL questionnaires and neurological tests. The study was approved by the local ethics committee and registered as a clinical trial (NCT04632797). Written informed consent was obtained from all patients prior to enrolment.

### 2.2 Patients

A total of 200 individuals were enrolled in this study. Patients who met the inclusion criteria were randomized 1:1 to receive either cryotherapy alone (C) or a combination of compression and cryotherapy (cryocompression, CC) on their upper extremities during chemotherapy treatment. Eligible patients were at least 18 years old, had a diagnosis of breast or other gynecological cancer and received a minimum of 3 cycles of taxane-based CT (neoadjuvant, adjuvant or palliative therapy). The exclusion criteria were preexisting polyneuropathy of grade 2 or above, neuralgia, bone or soft tissue metastasis in the hands or feet, Raynaud syndrome, cold intolerance, peripheral arterial ischemia or hand-foot syndrome. Polyneuropathy was assessed with the CTCAE score. This score distinguishes five grades of neuropathy: grade 1 (asymptomatic), grade 2 (moderate symptoms, limiting activities of daily living (ADL)), grade 3 (severe symptoms, limiting self-care), grade 4 (life-threatening consequences) and grade 5 (death) [19].

Since the effects of C and CC in preventing CIPN have previously been shown in clinical trials, we decided to not withhold treatment from a potential control group for ethical reasons. Instead, we decided to randomize all patients to receive one of the two options for preventing CIPN. During study planning, we assumed that cryocompression might be more efficient than cryotherapy, so the cryotherapy group was designed to be our control group.

### 2.3 Cooling and compression procedures

For patients in the C group as well as the CC group, cryotherapy or cryocompression started 30 min prior to the administration of a taxane-based CT. Cryotherapy and cryocompression were performed during the entire CT treatment and for an additional 30 min after its completion. Cooling in both groups was performed with Hilotherm® cooling devices. Patients with additional compression wore Sempermed® surgical gloves. Gloves that were one size smaller than the size that best fit their hands were worn under the cooling devices.

### 2.4 Study endpoints

The primary outcome was the occurrence of CIPN of grade 2 or above, which was compared between the two groups. The secondary outcome was the patients’ nail changes and patient satisfaction, which was measured with QoL questionnaires.

### 2.5 Assessment of polyneuropathy

Patients were assessed at five time points during the study: before the start of chemotherapy treatment (T0), mid-chemotherapy (T1), at their last chemotherapy cycle (T2), three months after chemotherapy (T3) and six to nine months after chemotherapy (T4). Adverse events were evaluated with the Common Terminology Criteria for Adverse Events score (CTCAE score). After each therapy session, redness and blistering were documented.

Polyneuropathy was assessed with the CTCAE score. The CTCAE score quantifies the severity of CIPN in sensory and motor components [19]. Since polyneuropathy of grade 2 or above has a clinically relevant impact on patients' lives, it is often used as a threshold to consider a dose reduction. Thus, in our study, CIPN of grade 2 or above was the decisive factor for our primary endpoint.

Before and after every cryotherapy or cryocompression session, the temperature of the fingertip of the index finger and the back of the hand was measured with the Exergen TemporalScannerTM Thermometer TAT 500®.

In addition, two neurological tests were conducted to examine potential sensory deficits at the distal phalanx of the index finger: the Rydell-Seifer test and the Semmes‒Weinstein monofilament test. The sensory Rydell-Seifer test provides a score that ranges from 0 to 8. A score of 8 is interpreted as no deficits in recognizing vibration. The Semmes‒Weinstein monofilament test provides a score that ranges from 3 to 19. A score of 3 indicates whether the smallest possible prick is recognized by the patient. The higher a patient's score on the Semmes‒Weinstein monofilament test is, the more sensory deficits are present. All tests and assessments were performed by a research team member on the patients' right upper extremity unless factors such as a prior injury could impact the test result; then, the left side was assessed.

The patient-reported outcomes included two questionnaires: the European Organization for Research and Treatment of Cancer (EORTC) Quality of Life CIPN 20 (EORTC QoLCIPN-20) and the Neuro-QoL Domain for Upper Extremity (Fine Motor domain, ADL). The EORTC Questionnaire measures CIPN symptoms and functional limitations associated with CIPN. The lowest possible score (18 or 19 depending on whether the patient drives a car) means that a patient's quality of life has not been highly affected by polyneuropathy. The Neuro-QoL Domain for the Upper Extremities contains questions regarding motor deficits. It consists of 20 questions about patients' ADLs, and the total score ranges from 20 to 100. The highest score of 100 means that ADL are not affected by CIPN.

Photographs of the patients’ fingernails were taken by a professional photographer at each checkup. Then three specialists evaluated nail changes independently using the CTCAE scale (grade 1 = discoloration, ridging or pitting; grade 2-partial or complete loss of nail(s)) [19].

### 2.6 Statistical analysis

Analyses were performed using SPSS version 29. To avoid imbalances both groups were compared using linear mixed models with adjustment for age, chemotherapy regimen and tumor type as these factors are linked to a higher risk of CIPN [20]. The planned sample size required a minimum of 175 patients to achieve a power of 80 % and a one-sided test with a significance level of 0.05 to dismiss the null hypothesis. All analyses were performed according to the intention-to-treat principle and were not otherwise specified. The primary endpoint was calculated using a chi-square test. The secondary endpoints were calculated using the Pearson Chi^2^ test and the Wilcoxon signed-rank test.

## 3 Results

### 3.1 Study population

**Fig. 1:**
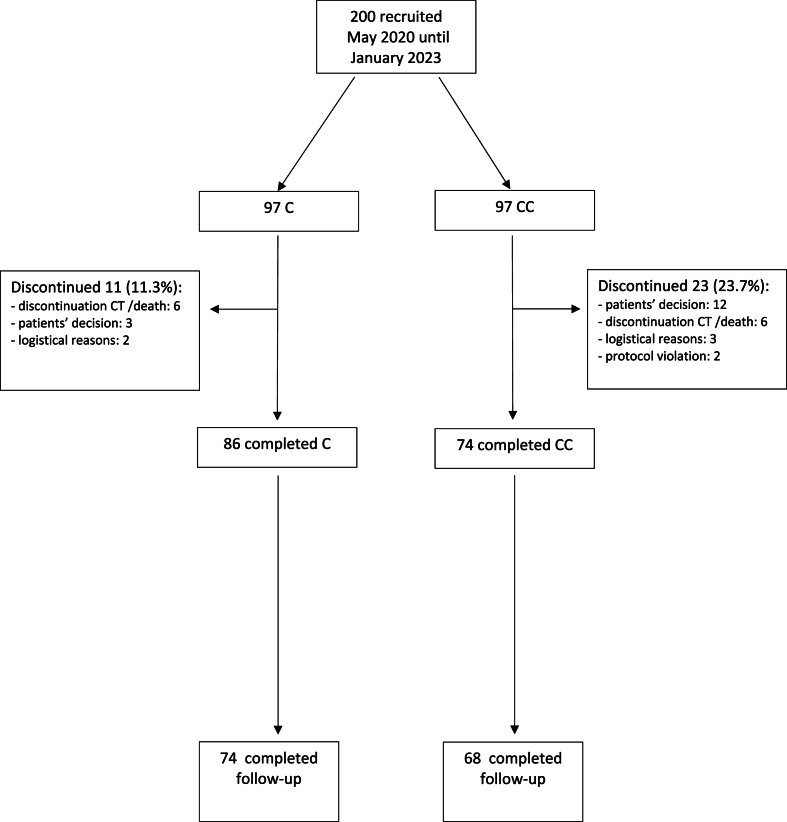
Consort diagram. C = cryotherapy; CC = cryocompression.

A total of 200 patients were enrolled in this study (Fig. 1). Three randomization mistakes occurred in each arm. Overall, 97 patients were randomized to the C group, and 97 patients were included in the CC group. A total of 86 patients in the C group and 74 patients in the CC group completed chemotherapy. Follow-up was completed by 86 % of patients in the C group and 92 % of patients in the CC group. Patient characteristics are shown in Table 1

**Table 1:** Patient characteristics.

	C n = 97	CC n = 97	ALL n = 194	p value
**Age (ys): median (range)**	52 (26–83)	55 (29–85)	54 (26–85)	0.253^a^
**Menopausal status**				0.04^b^
premenopausal	44 (45.4)	28 (28.9)	72 (37.1)	
postmenopausal	52 (53.6)	65 (67)	117 (60.3)	
unknown	1 [1]	4 (4.1)	5 (2.6)	
**BMI (median)**	24.1	24.1	24.1	
**Tumor features**				0.898^b^
breast cancer	75 (77.3)	76 (78.4)	151 (77.9)	
ovarian cancer	18 (18.6)	16 (16.5)	34 (17.5)	
other	4 (4.1)	5 (5.1)	9 (4.6)	
**Setting CT**				0.599^b^
neoadjuvant	52 (53.6)	49 (50.5)	101 (52.1)	
adjuvant	30 (30.9)	36 (37.1)	66 (34)	
palliative	15 (15.5)	12 (12.4)	27 (13.9)	
**Duration CT median**				
cycles	6	6	6	
**CT regimen**				0.814^b^
taxane mono	15 (15.5)	18 (18.5)	33 [17]	
T + A	59 (60.8)	57 (58.8)	116 (59.8)	
taxane + platinum	23 (23.7)	22 (22.7)	45 (23.2)	

C = cryotherapy; CC = cryocompression; CT = chemotherapy; T + A = taxane and anthracycline CT; ys = years.

a Mann-Whitney-U-Test.

b Chi2 -Test.

### 3.2 Prevalence of CIPN ≥ grade 2 during chemotherapy and follow-up

At the start of the study, over 95 % of patients in both groups had grade 0 polyneuropathy. At the end of the last CT cycle (T2), 30.1 % of the patients in the C group had grade 1 and 13.7 % had grade 2 or above polyneuropathy. In the CC group, 32.8 % of patients had grade 1 and 17.2 % at least grade 2 polyneuropathy.

At the first follow-up three months post-CT (T3), 30.1 % of the patients in the C group presented with grade 1 and 19.2 % presented with at least grade 2 polyneuropathy. Compared to the CC group, where 25.4 % had grade 1 and 29.8 % had grade 2 or above polyneuropathy.

**Fig. 2:**
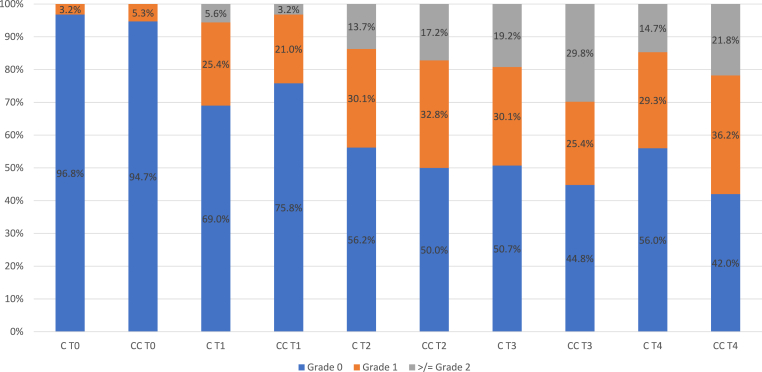
CTCAE CIPN during chemotherapy and follow up. C = cryotherapy; CC = cryocompression; T0 = start of CT, T1 = midway CT; T2 = end of CT; T3 = 3 months after CT, T4 = 6–9 months after CT.

Between T3 and T4, the number of patients with at least grade 2 polyneuropathy decreased in both groups. At T4 in the C group, 29.3 % of the patients had grade 1 and 14.7 % had grade 2 or above polyneuropathy. In the CC group, 29.3 % of the patients had grade 1 polyneuropathy and 21.8 % had grade 2 or above polyneuropathy. (see Fig. 2)

### 3.3 Comparison of efficacy of the two therapies

There was no statistically relevant difference between the two groups concerning the occurrence of grade 2 or above CIPN (Chi^2^ Test p-value: T1 = 0.313; T2 = 0.727, T3 = 0.589, T4 = 0.295)

### 3.4 Sensory tests and QoL questionnaires

The development of the sensory test and the QoL questionnaires are shown in Table 2. Regarding both neurological tests as well as both QoL questionnaires, there was no significant difference between the two groups (all p-values >0.1).

**Table 2:** Median Score sensory tests and QoL questionnaires.

	T0	T1	T2	T3	T4
**Rydell-Seifer test C**	8	8	7.5	8	8
**Rydell-Seifer test CC**	7.5	8	8	8	8
**Semmes‒Weinstein test C**	4	6	6	6	5
**Semmes‒Weinstein test CC**	3	6	6	6	6
**EORTC questionnaires C**	20	24	27	30	28
**EORTC questionnaires CC**	21	28	34.5	32	31
**Neuro-QoL questionnaires C**	100	100	100	100	100
**Neuro-QoL questionnaires CC**	100	100	100	100	100

C = cryotherapy; CC = cryocompression, T0 = start of CT, T1 = midway CT; T2 = end of CT; T3 = 3 months after CT, T4 = 6–9 months after CT.

### 3.5 Nail changes

**Fig. 3:**
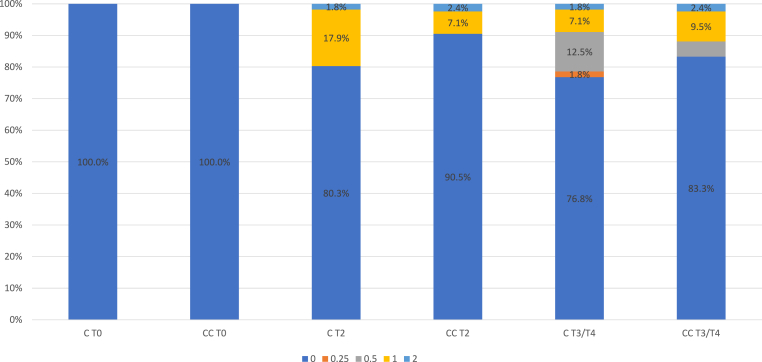
CTCAE nail changes during chemotherapy and follow up. C = cryotherapy; CC = cryocompression; T0 = start of CT, T1 = midway CT; T2 = end of CT; T3/T4 = median of follow-ups.

Overall nail changes were evaluated in 56 individuals in the C group and 42 in the CC group had. Documentation of nail changes took place at T0, T2 and T3/T4 (Median follow-up = Median of T3 and T4). An overview of the nail changes is provided in Fig. 3. There was no significant difference between the two groups (Chi^2^ Test p-value: T2 = 0.3; T3/4 = 0.618)

### 3.6 Skin temperature

The median skin temperature of the back of the hand and the index finger for both groups is shown in Table 3. Both groups showed a significant reduction in skin temperature. There was no significant difference between the two methods (p = 0.564 for the back of hand; p = 0.566 for the index finger).

**Table 3:** Cooling time and temperature.

	C (n = 97)	CC (n = 97)	ALL (n = 194)	p-value
**Cooling time (minutes)**	130	125	129	0.666
**Median temperature index finger (°C)**				
before cooling	35.8	35.9	35.9	0.568
after cooling	23.9	24.2	24	0.566
difference median	−11.9	−11.4	−11.7	0.151
**Median temperature back of hand (°C)**				
before cooling	35.8	35.8	35.8	0.551
after cooling	23.5	22.7	23	0.564
difference median	−12.4	−12.7	−12.5	0.774

C = cryotherapy; CC = cryocompression.

### 3.7 Adverse events

No patients withdrew from the study due to adverse events caused by cryotherapy or cryocompression. After each C or CC application, the patients were specifically checked for redness and blistering by the administering nurse.

In the C group, 71 % of the patients never developed redness, 20.4 % developed redness once and 8.6 % developed redness at least twice. In the group receiving CC, redness did not occur in 53.3 % of the patients; it occurred once in 32.6 % and at least twice in 14.1 %. Regarding blistering, neither group had any patients who experienced this adverse event twice or more times. In each group, one patient presented with blistering once (1.1 %).

### 3.8 Discontinuation of therapy

In the C group, 11.3 % of patients discontinued therapy, and in the CC group, 23.7 % discontinued therapy. The reasons for discontinuation are shown in Table 4. A significantly higher number of patients receiving cryocompression discontinued therapy compared to patients receiving cryotherapy (p = 0.0234).

**Table 4:** Reasons for discontinuation.

Reason for discontinuation	C	CC	ALL
**patients‘ decision**	3	12	15
**discontinuation chemotherapy or death**	6	6	12
**logistical reason**	2	3	5
**protocol violation**	0	2	2
**total**	11	23	34

C = cryotherapy; CC = cryocompression.

## 4 Discussion

One of the major findings of our study is that patients in both groups developed remarkably rarely CIPN of grade 2 or above. At the end of chemotherapy (T2), 13.7 % of the patients in the C group presented with polyneuropathy of at least grade 2, and in the CC group, 17.2 % did so. Moreover, regarding any grade of CIPN, our study had a significantly lower prevalence than recent studies. In the cryotherapy group, 43.8 % of the patients had CIPN, and in the cryocompression group, 50 % had CIPN. According to Oneda et al. and Jia et al. the incidence of CIPN related to taxane treatment is reported to be as high as 87 % [15,[21], [22], [23]]. A review by Ibrahim et al. states that up to 70 % of patients are affected by CIPN [23].

Our results are similar to those published by Kanbayashi et al., who aimed to compare cryotherapy to compression by treating patients with a frozen glove on one hand and a surgical glove on the other hand. The incidence of CIPN grade 2 or above was 18.4 % in both groups [6]. Likewise, in the POLAR study by Michel et al., with a similar study set up. The prevalence of CIPN grade 2 or above was 29 % in the cooling group and 24 % in the compression group [24].

Another relevant finding of our study is that the occurrence of CIPN lagged after the completion of CT. From the end of CT to the first follow-up visit, we found an increase in the percentage of patients with CIPN grade 2 or above. At the second follow-up six to nine months after the end of CT, the percentage of patients with CIPN grade two or above began to decrease to 14.7 % in the cryotherapy group and 21.8 % in the cryocompression group. This has been previously mentioned in literature as “coasting” and describes the phenomenon where patients’ CIPN continues to progress and deteriorates for 2–6 months after the cessation of CT [5,25].

In our study, there was no significant difference between the cryotherapy and cryocompression groups regarding the incidence of CIPN, the QOL questionnaire scores or the sensory test scores. The hypothesis that the benefits of a combination of

cryotherapy and compression is even more effective to prevent CIPN even more effectively, as reported by Bandla et al. [17], could not be confirmed.

Cryotherapy alone has been shown to be effective in the of CIPN in previous studies. In a study published by Shigematsu et al., patients receiving cryotherapy had a significantly lower incidence of CIPN of grade two or above and lower neurotoxicity scores [13]. Similarly, Chitkumarn et al. and Hanai et al. reported successful prevention of CIPN in the group treated with cryotherapy compared to their control group [14,16]. All three trials used frozen gloves.

Regarding compression alone to prevent CIPN, the current literature showed mixed results. Kotani et al. evaluated the use of surgical gloves to prevent CIPN but found no significant benefit compared to the control group [18]. However, Tsuyuki et al. reported that the occurrence rates of CIPN of grade two or above and sensory or motor peripheral neuropathies were significantly lower for the hands treated with compression compared to the control hands [26]. The previously mentioned promising results from the POLAR trial were presented at the ESMO 2022 and therefore could not be taken into consideration during the conception of our study in 2019 [24]. The authors admit that adding an arm with only compression would have certainly increased this study's value. But due to the limited literature available at the time in addition to our predicted patient numbers and limited resources the decision was made to focus on the comparison of cryotherapy vs. cryocompression therapy.

Current literature regarding cryocompression is extremely scarce. Bandla et al. reported successful prevention of CIPN with CC. None of their patients developed grade two CIPN [17]. In the study by Bandla et al., cryocompression led to a significantly lower skin temperature decrease of 2.32 °C compared to continuous-flow cooling [17]. In our study, we also measured temperature before and after treatment. Both C and CC led to a significant reduction in temperature in the patients’ extremities, but there was no significant difference between the two methods. Thus, we did not find an additional cooling effect by using compression in addition to cryotherapy. The effect that compression might isolate and cause a rise in temperature was not observed in our study. Our study used a surgical glove for compression, whereas Bandla et al. used cyclic pressure and reported better results [17]. Therefore, it seems that the application of cyclic pressure is the superior method for compression therapy.

Our study examined one of the largest patient populations with regards to CIPN. Nevertheless, our study had so many different CT protocols enrolled, that it was not possible to evaluate the impact of each CT on its own. Since CIPN incidence and severity vary according to the cumulative dose, duration of exposure, and scheduling and combination of different agents [7], larger multicenter studies are necessary to assess the impact of these factors and to identify other factors affecting the occurrence of CIPN.

One of the major limitations of all the studies regarding CIPN is the lack of standardized clinical measurement and, therefore, a lack of comparability [27]. By reducing the evaluation of CIPN to the CTCAE score might not do justice to the complexity of the adverse events. Especially for grade 2 CIPN, it would be more precise to further define the extend on how much patients' ADLs are affected. More precise scores to evaluate CIPN are needed to enable clinicians to realistically and distinctly evaluate a patient's CIPN.

## 5 Conclusions

Our study suggests that cryotherapy as well as cryocompression is a safe way to cool patients’ extremities to prevent CIPN. When compared to current literature patients treated with cryotherapy and cryocompression developed less CIPN. Furthermore, in our patient population, cryocompression did not seem to be more effective in cooling or preventing CIPN but led to a higher rate of therapy discontinuation due to discomfort.

## Ethical approval

We read and complied with the policy of the journal on ethical consent. The study was approved by the local ethics committee and registered as a clinical trial (NCT04632797). Written informed consent was obtained from all patients prior to enrolment.

## CRediT authorship contribution statement

**Christine Brunner:** Writing – original draft, Supervision, Project administration, Methodology, Formal analysis, Conceptualization. **Miriam Emmelheinz:** Writing – original draft, Investigation, Formal analysis, Data curation. **Daniel Egle:** Writing – review & editing, Investigation. **Magdalena Ritter:** Writing – review & editing, Investigation. **Katharina Leitner:** Writing – review & editing, Investigation. **Verena Wieser:** Writing – review & editing, Validation. **Carmen Albertini:** Writing – review & editing, Project administration. **Samira Abdel Azim:** Writing – review & editing, Validation. **Irene Mutz-Dehbalaie:** Writing – review & editing, Investigation. **Johanna Kögl:** Writing – review & editing, Investigation. **Christian Marth:** Writing – review & editing, Validation, Supervision, Formal analysis, Conceptualization.

## Declaration of competing interest

The authors declare the following financial interests/personal relationships which may be considered as potential competing interests:

Christine Brunner reports that financial support and administrative support were provided by Hilotherm (Germany) and a relationship with Hilotherm (Germany) that includes funding grants. Christine Brunner reports receiving consulting fees from Gilead Sciences and Novartis, honoraria for presentations from AMGEN, DaiichiSanyko, AstraZeneca, Gilead Sciences, SEAGEN, Pfizer and Novartis and support for attending meetings from DaiichiSanyko, AstraZeneca, and Novartis and participation on advisory board for Pfizer.

Miriam Emmelheinz reports receiving support for attending meetings from Teva Ratiopharm and Sandoz.

Daniel Egle reports receiving consulting fees from AstraZeneca, Daiichi-Sankyo, Gilead, Lilly, MSD, Novartis, Pfizer, Roche, Sandoz and Seagen and honoraria for presentations from Amgen, AstraZeneca, Daiichi-Sankyo, Gilead, Lilly, MSD, Novartis, Pfizer, Pierre-Fabre, Roche, Sandoz and Seagen and support for attending meetings from DaiichiSanyko, Gilead, Pfizer and AstraZeneca.

Katharina Leitner reports receiving support for attending meetings from Gilead, GSK, Eisai and Roche.

Verena Weiser reports receiving honoraria for presentations from Roche and Novartis and support for attending meetings from Roche.

Christian Marth reports receiving consulting fees from Amgen, AstraZeneca; GlaxoSmithKline, MSD, Novartis, PharmaMar, Roche and Seagen and honoraria for presentations from Amgen, AstraZeneca, GlaxoSmithKline, MSD, Novartis, PharmaMar, Roche and Seagen and support for attending meetings from AstraZeneca and Roche.

All other authors declare no conflicts of interest.
